# Efficacy and safety of sitagliptin added to metformin and insulin compared with voglibose in patients with newly diagnosed type 2 diabetes

**DOI:** 10.6061/clinics/2019/e736

**Published:** 2019-04-19

**Authors:** Chunhong Shi, Ru Zhang, Ran Bai, Dan Liu, Yongbo Wang, Xueyang Zhang, Hao Wang, Jianling Du

**Affiliations:** Department of Endocrinology, The First Affiliated Hospital of Dalian Medical University, Dalian, PR China

**Keywords:** Diabetes Mellitus, Type 2, Sitagliptin Phosphate, Voglibose, Combined Therapy, Efficacy, Safety

## Abstract

**OBJECTIVE:**

To assess the efficacy and safety of sitagliptin compared with voglibose added to combined metformin and insulin in patients with newly diagnosed type 2 diabetes (T2DM).

**METHODS:**

In this 12-week prospective, randomized, parallel trial, 70 newly diagnosed T2DM patients with glycosylated hemoglobin (HbA1c) ≥9% and/or fasting plasma glucose (FPG) ≥11.1 mmol/L were randomized (1:1) to receive sitagliptin 100 mg per day + metformin + insulin glargine or voglibose 0.2 mg three times daily + metformin + insulin glargine. Change in HbA1c at week 12 was the primary endpoint.

**RESULTS:**

The mean baseline HbA1c was 11.0% in the patients. The changes in HbA1c from baseline were -6.00% in the sitagliptin group and -3.58% in the voglibose group, and the between-group difference was -2.42% (95% CI -1.91 to -2.93, *p*=0.02). The differences in FPG and homeostatic model assessment of β-cell function (HOMA-β) and the change in body weight between groups from baseline were -2.95 mmol/L (*p*=0.04), 43.91 (*p*=0.01) and -2.23 kg (*p*=0.01), respectively. One patient (2.9%) in the sitagliptin group and three patients (8.6%) in the voglibose group exhibited hypoglycemia.

**CONCLUSIONS:**

Sitagliptin added to combined metformin and insulin therapy showed greater efficacy and good safety regarding hypoglycemia in patients with newly diagnosed T2DM compared with voglibose.

## INTRODUCTION

The prevalence of diabetes in China is as high as 11.6% 1. The main cause of disability and death are complications of diabetes. Approximately 10% of the national health budget is expended on treating diabetes and its complications 2. Good blood glucose control is an important measure that can delay the development of diabetic complications.

For newly diagnosed type 2 diabetes (T2DM) patients, choosing an appropriate hypoglycemic treatment strategy is crucial to achieve the goal of lowering blood glucose levels stably and safely. For patients with glycosylated hemoglobin (HbA1c) greater than 9% or fasting plasma glucose (FPG) greater than 11.1 mmol/L, insulin should be chosen to reduce blood glucose levels quickly 2 and relieve the effect of hyperglycemia on apoptosis, dedifferentiation and transdifferentiation of islet beta cells, and recovery islet cell function 3. Metformin, the only first-line and antihyperglycemic therapy drug for T2DM, appears in many diabetes treatment guidelines around the world 2,4. Metformin can reduce hepatic glucose output, promote glucose uptake and utilization in peripheral tissue, and improve insulin resistance. Metformin combined with insulin targets two pathogenic aspects, insulin resistance and secretion defects in T2DM.

Voglibose primarily inhibits invertase and maltase and ultimately inhibits the degradation of disaccharides into monosaccharides 5. Voglibose monotherapy can decrease HbA1c levels by 0.5%∼1.4% 6. Dipeptidyl peptidase-4 (DPP-4) inhibitors delay the degradation of glucagon-like peptide-1 (GLP-1) and increase endogenous GLP-1 levels, thus promoting insulin secretion 7. For patients with a mean initial HbA1c of 7.8%, sitagliptin monotherapy decreased HbA1c by 0.7% compared with placebo in treatment-naive T2DM patients 8. Studies have shown that for T2DM patients with poor blood glucose control, sitagliptin monotherapy 9 or sitagliptin added to metformin 10 have a significantly stronger effect of reducing HbA1c levels than voglibose monotherapy or voglibose and metformin combined. For T2DM patients who already receive insulin treatment, addition of sitagliptin can lead to a significantly higher decrease in HbA1c than addition of voglibose 11,12. Our previous studies used continuous subcutaneous insulin injection combined with either sitagliptin or voglibose to treat newly diagnosed T2DM and confirmed that after two weeks of treatment, sitagliptin had a stronger effect on decreasing mean blood glucose, fasting blood glucose and glucose fluctuation 13.

At present, no studies have been carried out on the long-term efficacy of adding sitagliptin compared with voglibose to combined metformin and insulin therapy for the treatment of newly diagnosed T2DM patients experiencing high glucose toxicity. In the present study, we added sitagliptin or voglibose to combined metformin and insulin therapy for treating newly diagnosed T2DM patients with HbA1c≥9.0% and/or FPG≥11.1 mmol/L. Twelve weeks later, the efficacy and safety of these two treatments were compared and analyzed.

## MATERIALS AND METHODS

### Design and subjects

This study used a randomized, prospective, parallel design. A total of 83 newly diagnosed T2DM patients from the First Affiliated Hospital of Dalian Medical University were screened. Patients were diagnosed with T2DM within the past year according to the 2013 American Diabetes Association (ADA) criteria, age from 18 to 65 years and fasting FPG≥11.1 mmol/L and/or HbA1c≥9%. These patients had never taken oral hypoglycemic agents or received insulin treatment prior to their participation in the present trial.

The exclusion criteria were as follows: the presence of acute complications of diabetes, such as diabetic ketoacidosis or hyperosmolar hyperglycemic syndrome; severe cardiovascular or cerebrovascular events within the past 6 months; kidney damage (estimated glomerular filtration rate less than 60 ml/min·1.73 m^2^), or liver damage (alanine aminotransferase or aspartate aminotransferase 2.5 times more than the normal upper limit); the presence of a tumor, severe infection, or stress; a history of acute pancreatitis; or a history of gastrointestinal surgery.

The protocol was approved by the ethics committee of the First Affiliated Hospital of Dalian Medical University (Ethics References No: YJ-KY-FB-2015-02) and performed in accordance with the Declaration of Helsinki and good clinical practice guidelines. All patients provided written informed consent and then received screening.

### Randomization and masking

Using a computer-generated random number sequence, the patients were randomly and evenly divided into groups receiving sitagliptin 100 mg per day + metformin + insulin glargine or voglibose 0.2 mg three times daily + metformin + insulin glargine. The random numbers were stored in a closed opaque envelope. The researchers and patients were aware of the grouping and the drug interventions, but the investigators in charge of data collection and analysis were masked to the grouping and drug interventions.

### Procedures

All patients received diabetes health education, diabetes diets were provided by the nutrition department, and the patients were instructed to comply with a routine exercise plan.

Patients in the sitagliptin group took sitagliptin (100 mg, MSD, Hangzhou) 15 minutes before breakfast; patients in the voglibose group took voglibose (0.2 mg, Takeda, Japan) three times daily with meals. Metformin hydrochloride (0.5 g, SASS, Shanghai) was also orally administered three times daily with meals.

Subcutaneous injection of insulin glargine (Sanofi, France) was administered prior to bedtime. Insulin glargine was used at a dosage of 0.2 U·kg^-1^ d^-1^ initially. The amount of insulin glargine was adjusted based on the results of a finger-prick test. For patients who had fasting blood glucose (FBG)≥7 mmol/L for two consecutive days, insulin glargine was increased by 2 U. For patients who had FBG<5 mmol/L or showed hypoglycemia without obvious cause, the insulin glargine dose was decreased by 2 U, as described previously 14. Administration of insulin glargine was discontinued when the dose was ≤10 U/d.

Every two weeks, telephone follow-up was conducted to record the current amount of insulin intake, fasting and postprandial blood glucose levels, hypoglycemic events and fingertip blood glucose levels at that time. At baseline and three months, the HbA1c level was examined, a 75-g oral glucose tolerance test was conducted, and FPG, two-hour postprandial plasma glucose (2-h PPG), fasting C peptide (FCP) and 2-h C peptide (CP) were measured. In addition, body weight was measured.

C peptide test: After fasting overnight, patients consumed a mixture of 150 mL of warm water and 150 mL of 50% glucose solution over a five-minute period. Venous blood was drawn before and two hours after consumption of the mixture. Serum FCP and 2-h CP levels were examined by electrochemiluminescence. Morning FBG and 2-h PBG levels after three meals were monitored using a finger-prick test (ACCU-CHEK Performa, Roche, Germany) according to the instructions 15. FPG, liver function, kidney function, and blood lipids were measured by an automatic biochemical analyzer (HITACHI 7600-210, Japan), and HbA1c was determined using HPLC (VARIANT-II, Bio-Rad, CA, USA). Homeostatic model assessment of insulin resistance (HOMA-IR) and β function (HOMA-β) were calculated according to well-established methods 16.

Symptomatic hypoglycemia was defined as the occurrence of dizziness, palpitations and other symptoms of sympathetic nerve stimulation; biochemical hypoglycemia was defined as a finger-prick blood glucose <4.4 mmol/L.

### Outcomes

The change in HbA1c from baseline to week 12 was the primary endpoint. The changes in FPG, 2-h PPG, HOMA-IR, HOMA-β, and body weight at week 12 compared with baseline, and duration and dosage of insulin application were the secondary endpoints.

### Statistical methods

The standard deviation was assumed to be 0.6%, as described previously 9. If the difference in HbA1c between the sitagliptin group and voglibose group was 0.5%, 33 patients were required in each group (66 patients in total) to reach 90% power, permitting a 10% dropout rate; thus, a total of 70 patients were included in the randomization.

The analysis of outcomes was conducted in accordance with the principle of intention to treat.

Data that accorded with a normal distribution are expressed as the mean ± standard deviation (x̄±s), intergroup differences were compared by an independent samples *t*-test, and intragroup differences were compared by a paired sample *t-*test. All *p*-values were two-sided, and *p*<0.05 indicates a statistically significant difference.

## RESULTS

### Baseline information

**Figure 1:**
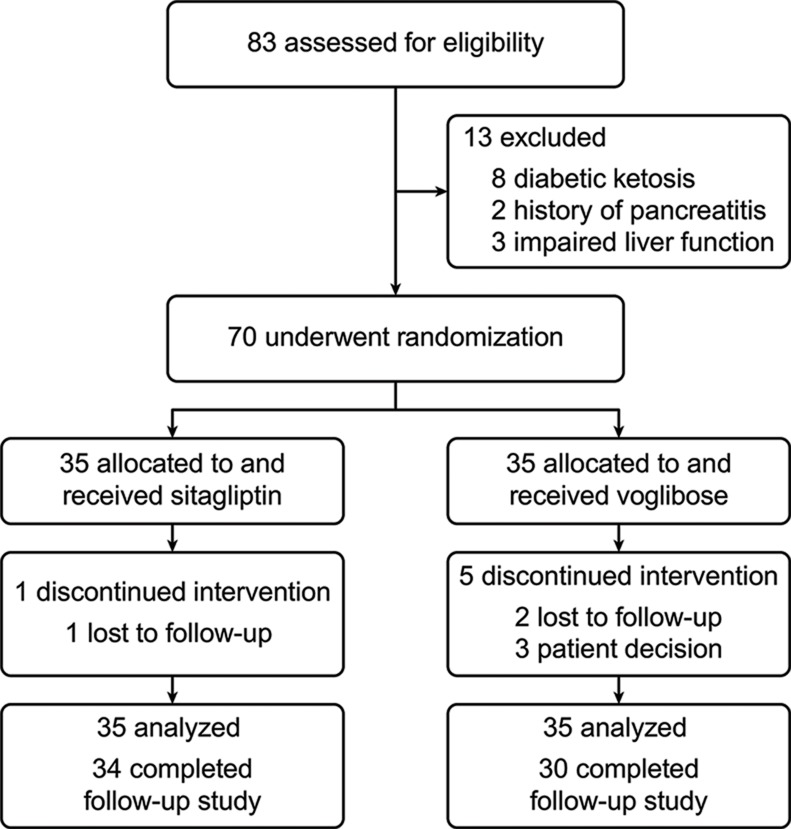
Enrollment.

From March 2015 to February 2016, 83 patients were screened, and 70 were enrolled and randomized into two groups. Six patients did not complete the 12-week follow-up (Figure 1). No statistically significant differences in initial characteristics were seen between the two groups (Table 1).

### Primary endpoint

**Figure 2:**
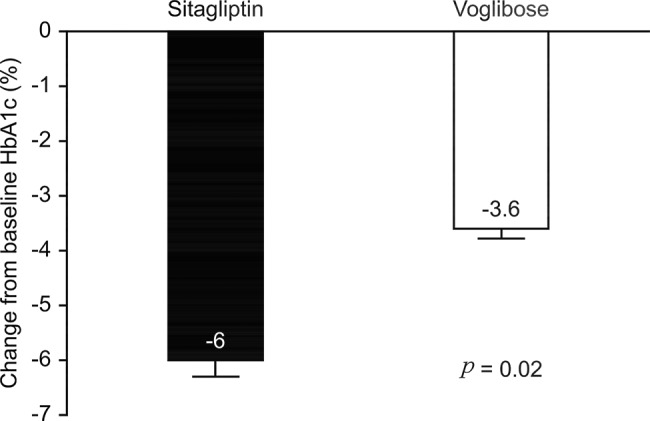
Comparison of HbA1c decline in the Sitagliptin and Voglibose groups after treatment.

At week 12, the initial changes in HbA1c were -6.00% in the sitagliptin group and -3.58% in the voglibose group. The difference between the groups was -2.42% (95% CI -1.91 to -2.93, *p*=0.02) (Figure 2).

### Secondary endpoints

**Figure 3:**
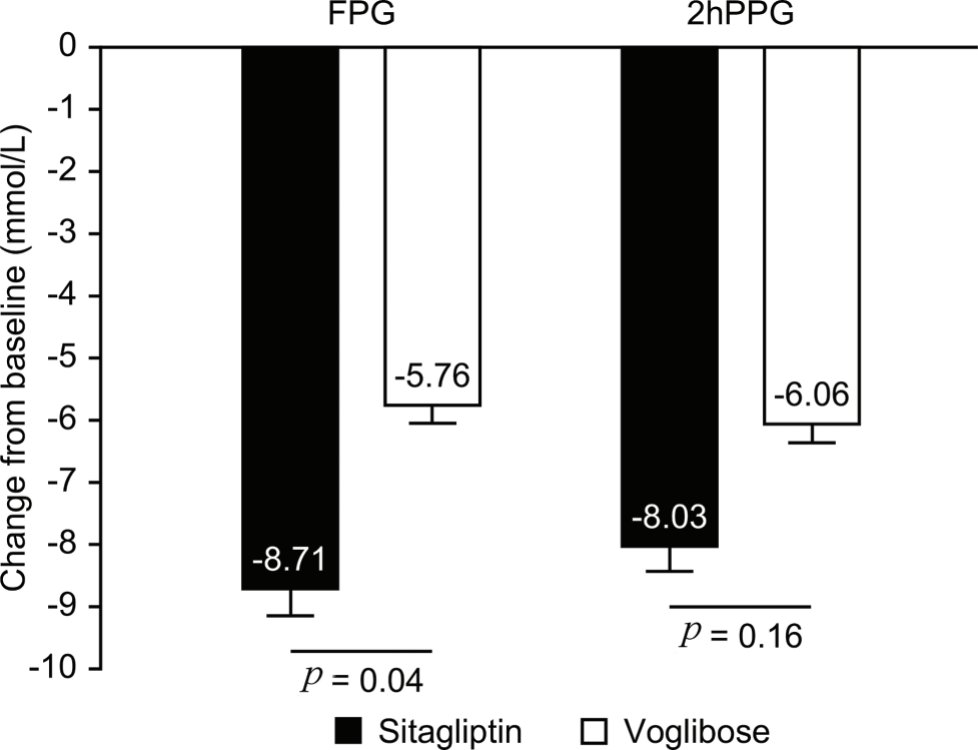
Comparison of FPG and 2-h PPG decline in the Sitagliptin and Voglibose groups after treatment.

After treatment, the decrease in FPG in the sitagliptin group (-8.71±2.99 mmol/L) was significantly greater than that in the voglibose group (-5.76±4.04 mmol/L), the difference was -2.95 mmol/L (*p*=0.04), although the decrease in 2-h PPG between the groups was not significantly different (*p*=0.16) (Figure 3).

**Figure 4:**
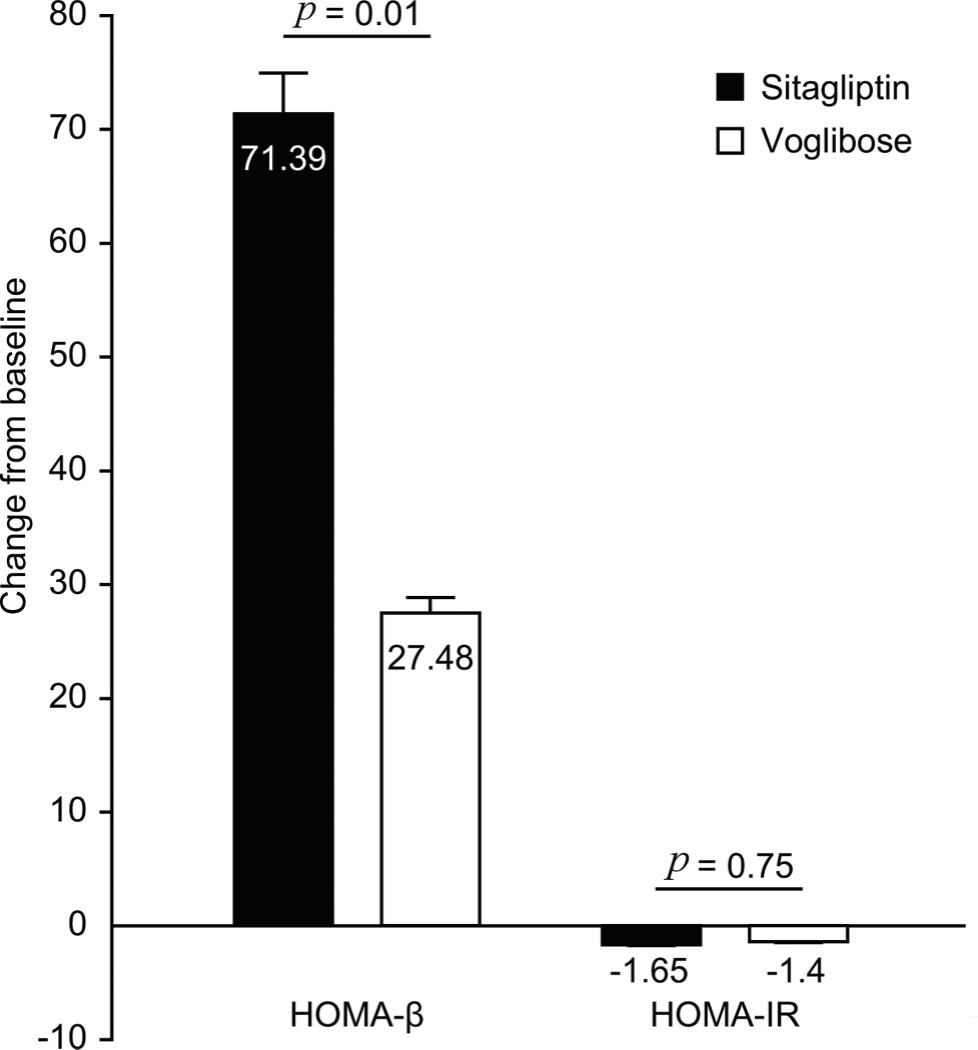
Comparison of HOMA-β and HOMA-IR change from baseline between the Sitagliptin and Voglibose groups after treatment.

After treatment, the increase in HOMA-β in the sitagliptin group (71.39±55.53) was significantly greater than that in the voglibose group (27.48±25.14), with a difference of 43.91 (*p*=0.01). The decrease in HOMA-IR between the groups was not significantly different (*p*=0.75) (Figure 4).

**Figure 5:**
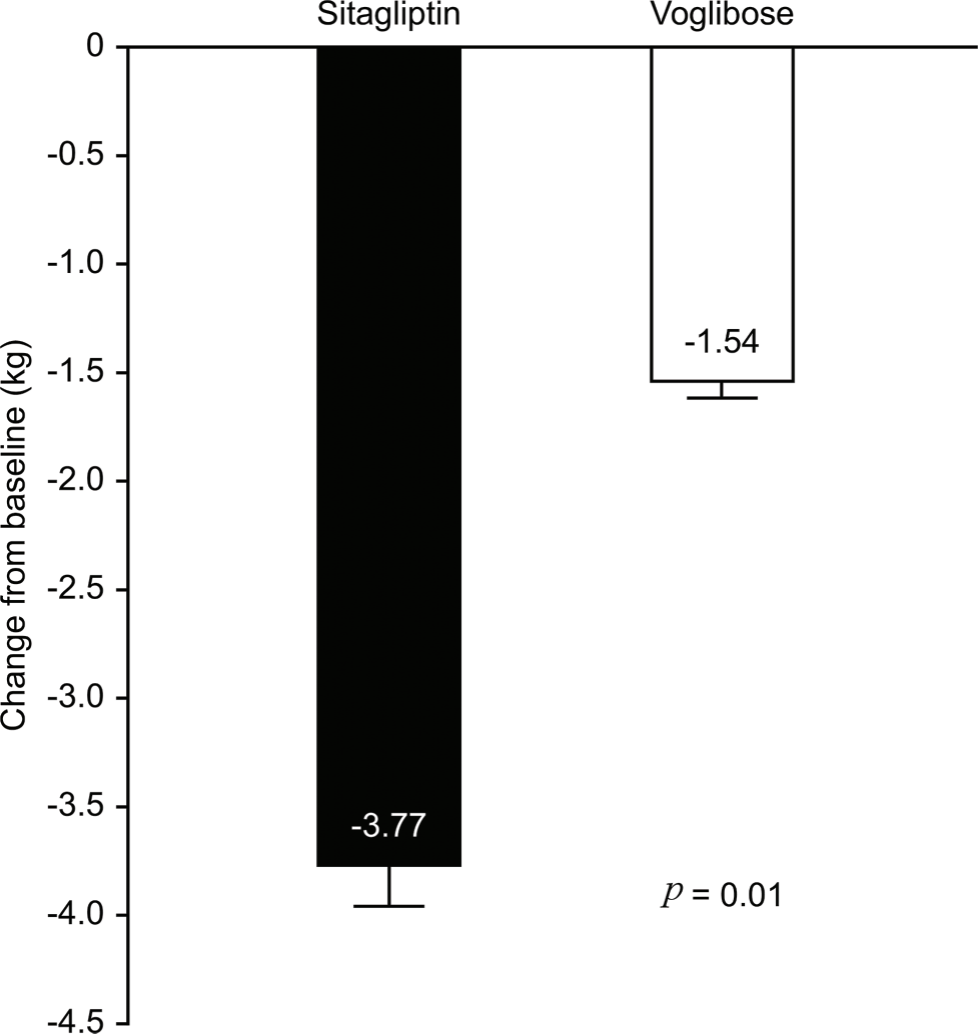
Comparison of weight change between the Sitagliptin and Voglibose groups after treatment.

After 12 weeks, weight loss in the sitagliptin group (-3.77± 1.88 kg) was significantly greater than that in the voglibose group (-1.54±1.05 kg), with a difference of -2.23 kg (*p*=0.01) (Figure 5).

The duration of insulin glargine use in the sitagliptin group (32±15 d) was significantly shorter than that in the voglibose group (50±27 d), with a difference of -18 d (*p*=0.04). The amount of insulin glargine administered was not significantly different between the groups (sitagliptin group 0.28±0.08 U·kg^-1^ ·d^-1^ *vs*. voglibose group 0.31±0.05 U·kg^-1^ ·d^-1^) (*p*=0.55).

### Safety endpoint

One patient in the sitagliptin group (2.9%) and three patients in the voglibose group (8.6%) exhibited symptomatic hypoglycemia. No incidents of biochemical hypoglycemia or nocturnal hypoglycemia occurred in patients in either group.

## DISCUSSION

For newly diagnosed T2DM patients with high blood glucose levels, intensive insulin therapy can quickly relieve high glucose toxicity and improve islet cell function. In this study, sitagliptin and voglibose added to insulin glargine combined with metformin were compared in a 12-week treatment regimen of a group of T2DM patients with high blood glucose levels. The results showed that the decreases in HbA1c and FPG in the sitagliptin group were significantly higher than those in the voglibose group, and the decrease in 2-h PPG between the groups did not differ. This result might have occurred because DPP-4 inhibitors reduce the degradation of glucagon-like peptides in the body, thereby increasing glucose-dependent insulin secretion, inhibiting glucagon secretion, and ultimately lowering fasting and postprandial blood glucose levels 17. Alpha glucosidase inhibitors (AGIs) reduce postprandial blood glucose primarily by reducing intestinal carbohydrate absorption. Therefore, sitagliptin is superior to voglibose for lowering fasting blood glucose levels when either is combined with insulin and metformin.

Since sitagliptin was superior to voglibose for lowering fasting blood glucose levels, even though no significantly different effects were seen on postprandial blood glucose levels between the groups, sitagliptin was also more effective than voglibose in reducing HbA1c. A similar conclusion was reported in a previous study. Compared with AGIs, the efficacy of sitagliptin in patients with poorly controlled T2DM compared with that of stable glimepiride alone (mean HbA1c 7.7%) was assessed after 12 weeks. Notably, HbA1c was reduced by -0.44% in the sitagliptin group (*p*<0.001) 18. A meta-analysis confirmed that changes in HbA1c (weighted mean deviation -0.30%; 95% CI -0.47 to -0.13%, *p*<0.001) and FPG levels (weighted mean deviation -0.50 mmol/L; 95% CI -0.89 to -0.11 mmol/L, *p*=0.01) were significantly greater in the DPP-4 inhibitor treatment group compared with that in the AGI group 19.

The present study found that HOMA-β in the sitagliptin group increased significantly after 12 weeks, suggesting a significant improvement in islet function. In a double-blind, crossover clinical trial, Tremblay et al. 20 found that T2DM patients receiving sitagliptin therapy displayed significantly improved islet function compared with patients receiving a placebo. In the present study, the voglibose treatment group also showed improved islet cell function and reduced insulin resistance, consistent with the results reported by Do et al. 21. The increase in HOMA-β in the sitagliptin group was significantly greater than that in the voglibose group. This finding suggests that sitagliptin more effectively protected and improved islet cell function, possibly because DPP-4 inhibitors inhibit the degradation of incretin, which has an indirect role in the inhibition of pancreatic β-cell apoptosis, and promote the proliferation of β cells 22. Voglibose relieves only high glucose toxicity to pancreatic β cells; therefore, compared with the voglibose group, the sitagliptin group showed better islet β cell protection.

In patients with newly diagnosed T2DM, glucotoxicity and lipotoxicity could be effectively reduced by early insulin therapy to alleviate insulin resistance and protect islet function 23. After short-term insulin therapy, patients with highly elevated blood glucose levels can gradually reduce or discontinue insulin treatment. In the present study, when the insulin glargine dose was reduced to less than 10 U/d and blood glucose was still well controlled, administration of insulin glargine was discontinued. The duration of insulin glargine use in the sitagliptin group (mean 32 d) was significantly shorter than that in the voglibose group (mean 50 d). This result might be due to a better effect of sitagliptin on lowering blood glucose and improving islet function; therefore, less insulin glargine was required to control blood glucose. The fact that there was no difference between the two groups in the average insulin dose per day per kilogram of body weight indicates that the shorter period of insulin use required in the sitagliptin group compared with that in the voglibose group was not due to the insulin dose.

Weight loss can improve insulin sensitivity; therefore, weight loss is essential for obese T2DM patients. The present study showed significant weight loss in both groups after treatment. Compared with placebo, DPP-4 inhibitors 24 and AGIs 25 can reduce body weight, consistent with the conclusions of our study. In addition, the sitagliptin group displayed significantly more weight loss than the voglibose group. A prospective, randomized, multicenter study treated T2DM patients with sitagliptin (50 mg/d) or voglibose (0.6 mg/d) for 24 weeks, and weight loss with sitagliptin treatment was significantly higher than that with voglibose treatment (-1.3±3.2 kg *vs*. 0.4±2.8 kg) 26, consistent with the results of the present study.

Hypoglycemia increases cardiovascular risk and is a barrier to blood glucose control in patients with diabetes. Our study showed that the sitagliptin group had one occurrence and that the voglibose group had three occurrences of symptomatic hypoglycemia. No incidents of biochemical hypoglycemia or nocturnal hypoglycemia occurred in either group. Both treatments therefore resulted in a low incidence of hypoglycemia and showed excellent safety. In our previous study, we compared the incidence of hypoglycemia in newly diagnosed T2DM patients receiving combination treatment with continuous subcutaneous insulin injection and DPP-4 inhibitors or AGIs; two weeks of dynamic blood glucose monitoring showed that both therapies have excellent safety 12.

The limitations of our study are that the number of included patients was relatively small and the follow-up time was relatively short. The results therefore require verification in large-scale, multicenter clinical trials with more patients and longer follow-up times.

In conclusion, compared with voglibose, sitagliptin added to combined insulin and metformin therapy can achieve a significantly better effect on lowering HbA1c and FPG and shows excellent safety for newly diagnosed T2DM patients with highly elevated blood glucose.

## AUTHOR CONTRIBUTIONS

Shi C and Zhang R conducted the statistical analysis and wrote the manuscript. Liu D and Wang Y collected the data. Zhang X, Wang H and Du J participated in the design of the study and edited the manuscript. Bai R designed and directed the entire study, and revised the manuscript.

## Figures and Tables

**Table 1:** Baseline characteristics of the S Group and V Group (x̄±S).

	S Group (n=35)	V Group (n=35)
Sex (male/female)	20/14	18/16
Age (years)	41.2±10.0	43.1±12.0
Weight (kg)	76.15±13.57	76.72±9.36
WC (cm)	92.99±11.38	94.19±8.29
BMI (kg/m^2^)	26.23±3.45	26.86±2.57
SBP (mmHg)	130.26±8.35	131.33±12.61
DBP (mmHg)	82.75±5.50	82.26±8.47
FPG (mmol/L)	14.99±2.73	14.50±3.11
HbA1c (%)	11.9±1.5	10.6±1.1
TC (mmol/L)	5.54±1.36	5.16±1.04
TG (mmol/L)	2.17±1.54	1.73±0.88
HDL-C (mmol/L)	1.72±1.02	1.34±0.26
LDL-C (mmol/L)	3.32±0.98	3.26±0.74

S Group: Sitagliptin, V Group: Voglibose. WC: waist circumference, BMI: body mass index, SBP: systolic blood pressure, DBP: diastolic blood pressure, FPG: fasting plasma glucose, HbA1c: glycated hemoglobin A1c, TC: total cholesterol, TG: triglyceride, HDL-C: high-density lipoprotein cholesterol, LDL-C: low-density lipoprotein cholesterol.
